# Editorial: Biomarkers in genitourinary cancers: Volume II

**DOI:** 10.3389/fonc.2022.1048736

**Published:** 2022-10-04

**Authors:** Takeshi Yuasa

**Affiliations:** Department of Urology, Cancer Institute Hospital, Japanese Foundation for Cancer Research, Tokyo, Japan

**Keywords:** biomarker, immune checkpoint inhibitor, prostate cancer, urothelial cancer, renal cell cancer

Genitourinary cancers, which consist of prostate, renal, urothelial, and testicular cancers, are some of the leading causes of global mortality (Yuasa et al.). Recently, various new technologies have been implemented in clinical practice, including robot-assisted surgery, intensity-modulated radiation therapy (IMRT), and radionuclide therapy. In addition, various anti-cancerous agents, such as antibody drug conjugates (ADCs), poly ADP-ribose polymerase (PARP) inhibitors, and immune checkpoint inhibitors, have been adopted in clinical practice (Yuasa et al.). Based on their promising anti-tumor efficacy and manageable safety profile, the paradigm of medical treatment for patients with urological malignancies is changing dramatically (Yuasa et al.). Biomarkers have grown increasingly essential in the consideration of treatment strategies.

In *Biomarkers in Genitourinary Cancers: Volume II*, various candidate biomarkers are reported. (Yang et al., Wang et al., Shi et al. Jin et al., Gan et al., Lang et al., van Laar et al.). Yang et al., Wang et al., and Shi et al. stressed the importance of circulating tumor cells (CTCs) and circulating endothelial cells (CECs), systemic inflammation markers, and the cell cycle checkpoint–related genes signature as biomarkers for bladder cancer, respectively. Jin et al. reported urine exosomal AMACR as a candidate biomarker for prostate cancer detection at biopsy. Gan et al. demonstrated that ASNS may play a significant role in the development and immune cell infiltration of clear cell renal cell cancer. In contrast, in order to explore effective therapeutic agents for individual cases, Lang et al. developed preclinical patient-derived xenograft (PDX) models that recapitulate the molecular heterogeneity of urothelial cancer, including actionable mutations. It is unknown whether these factors will advance in clinical practice or remain candidates forever.

Various factors contribute to the survival outcome of patients with malignant disease. Tumor biology and aggressiveness, which include Gleason score for prostate cancer, Fuhrman nuclear grade for renal cell cancer, and tumor grade for urothelial cancer, have been major prognostic factors in clinical practice. Another important factor related to outcome is treatment efficacy and resistance. Indeed, van Laar et al. reported the alteration of treatment patterns and outcomes of metastatic RCC patients in the Netherlands (van Laar et al.). This factor depends on the treatment agent and may vary from day to day. In metastatic renal cell cancer, the sarcomatoid variant and high expression of PD-L1 were associated with poor outcome (1, 2). The introduction of immune checkpoint inhibitors may change this scenario (3–5). The biomarkers in this category may change as a result of revised therapeutic strategy. As such, the continued exploration of treatment-related biomarkers is important.

## Author contributions

TY contributed to conception and design of the study, wrote the first draft of the manuscript, and read, and approved the submitted version.

## Conflict of interest

The author declares that the research was conducted in the absence of any commercial or financial relationships that could be construed as a potential conflict of interest.

## Publisher’s note

All claims expressed in this article are solely those of the authors and do not necessarily represent those of their affiliated organizations, or those of the publisher, the editors and the reviewers. Any product that may be evaluated in this article, or claim that may be made by its manufacturer, is not guaranteed or endorsed by the publisher.
